# Access to and quality of sexual and reproductive health services in Britain during the early stages of the COVID-19 pandemic: a qualitative interview study of patient experiences

**DOI:** 10.1136/bmjsrh-2021-201413

**Published:** 2022-04-20

**Authors:** Raquel Bosó Pérez, David Reid, Karen J Maxwell, Jo Gibbs, Emily Dema, Christopher Bonell, Catherine H Mercer, Pam Sonnenberg, Nigel Field, Kirstin R Mitchell

**Affiliations:** 1 MRC/CSO Social and Public Health Sciences Unit, University of Glasgow, Glasgow, UK; 2 The National Institute for Health Research Health Protection Research Unit in Blood Borne and Sexually Transmitted Infections in partnership with Public Health England, University College London, London, UK; 3 Institute for Global Health, University College London, London, UK; 4 Faculty of Public Health and Policy, London School of Hygiene and Tropical Medicine, London, UK

**Keywords:** COVID-19, qualitative research, Health Services Accessibility, Reproductive Health Services, Sexual Health

## Abstract

**Introduction:**

Access to quality sexual and reproductive health (SRH) services remains imperative even during a pandemic. Our objective was to understand experiences of delayed or unsuccessful access to SRH services in Britain during the early stages of the COVID-19 pandemic.

**Methods:**

In October and November 2020 we conducted semi-structured telephone interviews with 14 women and six men reporting an unmet need for SRH services in the Natsal-COVID survey, a large-scale quasi-representative web-panel survey of sexual health and behaviour during COVID-19 (n=6654). We purposively sampled eligible participants using sociodemographic quotas. Inductive thematic analysis was used to explore service access and quality and to identify lessons for future SRH service delivery.

**Results:**

Twenty participants discussed experiences spanning 10 SRH services including contraception and antenatal/maternity care. Participants reported hesitancy and self-censorship of need. Accessing telemedicine and ‘socially-distanced’ services required tenacity. Challenges included navigating changing information and procedures; perceptions of gatekeepers as obstructing access; and inflexible appointment systems. Concerns about reconfigured services included reduced privacy; decreased quality of interactions with professionals; reduced informal support; and fewer preventive SRH practices. However, some participants also described more streamlined services and staff efforts to compensate for disruptions. Many viewed positively the ongoing blending of telemedicine with in-person care.

**Conclusion:**

The COVID-19 pandemic impacted access and quality of SRH services. Participants’ accounts revealed self-censorship of need, difficulty navigating shifting service configurations and perceived quality reductions. Telemedicine offers potential if intelligently combined with in-person care. We offer initial evidence-based recommendations for promoting an equitable restoration and future adaption of services.

**Key messages**

- In describing their experiences of delayed or unsuccessful attempts to access SRH services, participants reported feeling hesitant and some self-censored their need.
- Challenges to accessing services included confusing and inconsistent information and procedures, and gatekeepers who were perceived as obstructive.
- Participants observed new efficiencies within services, including telemedicine, but also perceived reduced privacy and quality of interaction with providers.

## Introduction

Responding to rising COVID-19 cases, the UK announced a national lockdown in March 2020, imposing stay-at-home orders and discouraging non-essential contact including between healthcare workers and patients. To minimise SARS-CoV-2 transmission, providers rapidly adjusted how they delivered sexual and reproductive health (SRH) services through new protocols, staff redeployment and site closures.^1 2^ Early abortion care via telemedicine was an example of successful adaptability, reducing waiting times and barriers to access^3^ and increasing satisfaction.^4–6^ However, other essential SRH services such as long-acting reversible contraception (LARC) provision or asymptomatic sexually transmitted infection (STI) screening were halted or reduced.^1 7^ Providers tried to prioritise those with most need who could not be managed remotely for in-person care.^8^

Access to SRH services remains imperative even during a pandemic.^9^ People have a right to sexual expression, reproductive autonomy, safe childbirth and a life free from infection.^10^ The population need for services such as routine and emergency contraception, STI testing and treatment, sexual problems advice or antenatal care is continual. Disruptions to these services can have significant repercussions including unplanned pregnancy, undiagnosed STIs and sexual dysfunction.

The Natsal-COVID study sought to understand the initial impact of service reduction and reconfiguration in Britain (consisting of England, Scotland and Wales).^11^ SRH services in Britain are delivered by a range of providers including general practitioners (GPs), specialist integrated sexual health services and screening programmes (e.g., chlamydia or cervical cancer screening). Natsal-COVID Wave 1, undertaken in July–August 2020, is a quasi-representative web-panel survey designed to understand the early impact of the pandemic on SRH. The study highlighted unmet need for SRH services, with one in 10 survey participants reporting unsuccessful attempts to access SRH services and one in five men needing but being unable to access condoms.^12^ Other UK studies found that men who have sex with men and young people experienced an unmet need for STI testing, contraception and condom access.^13–15^ This paper describes the results from qualitative follow-up interviews with Natsal-COVID participants exploring the experiences of unmet or delayed SRH need in the general population. Learning from patient perspectives is crucial to inform recovery and rebuilding efforts during and after COVID-19.^8^

The study aimed to explore: (1) what challenges arose for patients attempting to access and navigate SRH services during the pandemic, and (2) how COVID-19 protocols and reduced staffing affected patient perceptions of service quality.

## Methods

### Study design and participants

Natsal-COVID is a mixed-method study exploring the impact of the COVID-19 pandemic on sexual behaviour, relationships and SRH.^11^ Following the Wave 1 web-panel survey, follow-up qualitative interviews were carried out to explore sexuality-related topics relevant during the pandemic. This paper draws on interviews with 20 of the 311 participants who, in the survey, reported unmet SRH access since lockdown and agreed to recontact. Quotas were applied to ensure variation by age, gender, ethnicity and region. We sought to include a minimum number of participants living in Scotland and Wales, and to oversample women to reflect their higher use of SRH services. Ethical approval was obtained from the University of Glasgow MVLS College Ethics Committee (20019174) and LSHTM Research Ethics Committee (22565).

### Data collection

The research team telephoned individuals who agreed to recontact, fell within the pre-specified quotas and provided valid contact details. An introductory call explained the study and confirmed eligibility. Those interested were emailed a study information sheet and given the opportunity to ask questions. Informed consent to participate was sought and recorded prior to interview. Interviews were conducted by three trained qualitative interviewers (DR, KJM and RBP) between 2 October 2020 and 16 November 2020. All interviews were conducted by telephone, lasting 45–90 min. The interview guide explored the context of help-seeking, experiences of attempting to access SRH services, impact of unmet or delayed need, and attitudes and experiences with telemedicine (see online supplemental material). Fieldnotes (summaries and reflections) were recorded after each interview. Participants were offered a £30 e-voucher for their time and contributions.

10.1136/bmjsrh-2021-201413.supp1 Supplementary data

### Data analysis

Audio recordings were professionally transcribed verbatim. Transcripts were reviewed by DR, KJM and RBP for accuracy and familiarity. Identifying details were removed. Data were thematically analysed^16^ to inductively identify themes pertinent to policy and practice. Participants’ partners’ experiences were occasionally related and were included in the analysis. Analysis was aided by NVivo 12 (QSR International), a CAQDAS software. DR open coded five transcripts, which involved inductively labelling data into discrete codes. These codes were reviewed by the analysis team and a draft coding frame was developed via discussion. To maximise coding consistency, DR and RBP double-coded three interviews, clarifying coding uncertainties and discrepancies to develop the final coding framework applied to the data. DR then summarised data from each transcript by category. Differences, similarities and range of experience were identified between and within cases. Interpretations of categories, themes and groupings were made by DR, RBP, KRM and KJM with summary explanations and quotes chosen.

## Results

Our sample included 14 women and six men (table 1). At the time of interview, 10 participants reported an ongoing unmet need, six had their needs partially met and four had met their needs after some delays. Participants commonly attempted to access more than one service. The most sought services were contraception (n=14), STI tests (n=6) and maternity/antenatal services (n=4). Our analysis is organised under four themes (see table 2 for supporting quotes).

**Table 1:** Sample characteristics

Gender	
Women	14
Men	6
Age	
18–29	3
30–39	10
40–49	7
Sexual identity	
Homosexual/gay	2
Heterosexual/straight	15
Omnisexual*	1
Not reported	2
Ethnicity	
Asian	3
Mixed	1
White	16
Region	
England	16
Scotland	3
Wales	1
SRH services	
Condoms and contraception	14
STI test	6
STI follow-up care	2
HIV prevention	1
HIV care	1
Sexual function and gynaecological problems	3
Fertility services	3
Advice or counselling for sexual problems	1
Maternity/antenatal services	4
Cervical screening	3
Outcome†	
Eventually met need	4
Partially met need	6
Unmet need	10

*The sexual attraction to all genders.

†Assessed by analyst based on interview.

**Table 2:** Examples of participant quotes supporting each theme

Theme	Example excerpt
(1) Hesitation and self-censorship	**Minimisation of one’s needs in comparison to others** *I didn't really want to go to the GP and bother them when other people might need to see somebody more than I do*. (F, 18–29)
	**Hesitation due to concerns about COVID-19** *I think I would feel more secure to stay home and not to go to a GP practice with people already … I guess now I am being more paranoid or more careful.* (F, 18–29)
	**Resignation after encountering barriers** *It just got too difficult to get hold of any (of the pill) again, I just gave up, and I thought, this isn't worth it. … It was too much effort, you know.* (F, 40–49) *I can’t get a referral … I was honestly going around in circles all the time. In the end because I was getting turned away all the time by the … on phone calls, I’d just think well what’s the point, there’s no point in me even ringing up. In the end I just suffered on really.* (F, 30–39)
(2) Navigating access to telemedicine and ‘socially-distanced’ services	**Incorrect information and contradictory procedures on service access** *I’d ring the phone numbers that were on the letters from previous … if you need to contact us, contact us on this number that type of thing. I’d ring and it would just ring and ring, and ring, and ring. I don’t know who to get in touch with.* (F, 30–39) *They said you should go and speak to the hospital. (And the hospital said) ‘There’s no phone appointment, only phone appointments are available in GP’. And GP said ‘This is not our department, contraception clinic is not our department, we can't say, we can't do anything, we can't prescribe … you have to go to hospital and speak to them’.* (F, 40–49) “*They kept messing her about saying to her, ‘come’, then ‘don’t come’, then ‘come’, then ‘don’t come’(to have a weight-check before accessing contraception)”* (M, 30–39)
	**Factors facilitating service access** *There’s a special ward for mums that are having problems. So, it’s like am emergency type of thing, so that was quite difficult to access … we’ve got family who are doctors, so they know how these work … So, we were able to understand what the system was like, but I think people who don’t have people in the know it must be a lot more difficult.* (M, 30–39)
	**Impact of encountering barriers to service access on choice and autonomy** *They sent me two packets of oral contraceptives through the post, which, to be fair, I never took, I just never took. Because that wasn’t what I was after, it wasn’t what I wanted, I just wanted my coil replaced. My coil has been great … it helped to level out my mood. … I just want a new coil*. (F, 40–49) *I was left helpless, stuck with menopause symptoms … it just made me feel like I didn’t matter* (F, 40–49).
(3) Experiencing telemedicine and ‘socially-distanced’ services	**Privacy during remote/‘socially-distanced’ consultations** *You have to talk through an intercom, from the outside (of the GP*). *And everybody now knows what’s wrong with you, … it’s on the street, as well, so anybody walking past can just hear what you're saying.* (F, 40–49)
	**Accessibility barriers to remote service access** *There’s always a bit of a delay on the video, and because it’s a bit stilted, and because I’m hard of hearing, I can’t always lip read. So when they’re asking me questions, sometimes, I’m completely in the dark about what they’re asking me*. (F, 40–49)
	**Impact of lone navigation of SRH services** *As good as all the staff are, if you're getting bad (pregnancy) news in a situation like that it is going to cause you a lot more stress if you don't have someone with you.* (F, 30–39) *He feels less involved (in the pregnancy*). *He’s not there to see and hear for himself sort of what’s going on. He’s having to sit and wait for me to relay everything.* (F, 30–39).
	**Positive experiences of remote/‘socially-distanced’ SRH services** *There were a few silver lining moments. We didn't have to wait that long to go into our appointment, the appointments themselves were a lot faster. We still got the good service from the midwives*. (F, 30–39) *They did all the tests there and then, but they had to send them off and then they phoned me, I think, a week later to say that everything was fine apart from the chlamydia which was positive and that they would … they posted the antibiotics out, so that was quite easy actually, it arrived in the post*. (F, 40–49)
	**Increased comfort during remote appointments** *The phone probably is easier because you’re not having to look at somebody and you don’t get the nervousness … if you’re suddenly kind of face-to-face with somebody that looks quite official, that might make you clam up.* (M, 30–39)
(4) Attitudes towards the continuation of telemedicine	**Increased convenience of telemedicine** *It (telemedicine) would just fit in better with day-to-day life. Because obviously, like modern life, you haven’t got a lot of time today now, you don’t really want to have to take time sat in a doctor’s surgery, you know, just to pick up some pills.* (M, 30–39) *I would totally like that (remote appointments) because sometimes for a (fertility) treatment you just have to do consent forms that you have to be there present but if they're done online that’s hopefully saved or like a few hours of your time. Sometimes just a brief discussion of what the process would be you don’t have to go to the clinic or the place. Yeah, these are really welcome changes from my point of view, and my partner as well.* (F, 30–39)
	**Limits to the suitability of telemedicine** *I feel like my personal privacy is not at its best at the moment in my current living situation. So, I’d rather nothing of that sort comes (referring to remote appointments or postal delivery*)*.* (M, 18–29) *We still need real people to be there and to assess because technology is not necessarily accessible to everybody.* (F, 18–29)

### Hesitation and self-censorship

Participants frequently discussed hesitation to use services, often linked to self-censorship of need. Many downplayed their needs relative to others, particularly people with COVID-19, and did not wish to burden an already pressured health system. While several mentioned being okay to postpone seeking healthcare, others felt sexual health was less of a priority for service providers during COVID-19 and that they too should de-prioritise it.

I didn’t even think it [condoms/STI tests] would be an important subject for them [SRH service], which probably it still might have been, but I think I felt like coronavirus is just ruling everything. (M, 18–29)

Other sources of hesitation to access healthcare included fear of contracting COVID-19 through clinic attendance and fear of healthcare providers’ disapproval for being sexually active when contact between households was restricted. One participant who was having condomless sex explained why he did not access condoms and STI testing:

[The clinic staff] might be like “Why would you need that, what have you been doing the last three months?” … I panicked that what if they contact a service and I get fined. (M, 18–29)

For some participants, hesitation led to inaction. Among those who did seek help, many waited to exhaust other alternatives or until their health needs worsened:

I'd left it for a while anyway, I think I wasn't entirely sure whether it was just a bit of thrush or a urine infection. By the time I figured out that it probably was chlamydia, then trying to get hold of somebody, that all dragged on. (F, 40–49)

Self-censorship did not always precede help-seeking. Several participants started to downplay their needs after encountering barriers to access (e.g., long queues or difficulty booking appointments). They resigned themselves to wait, believing they could *‘*grin and bear it’ and that the effort required would not achieve the expected benefit.

### Navigating access to telemedicine and ‘socially-distanced’ services

Participants found that accessing a healthcare professional took longer or proved harder than expected. Many SRH services were closed, on pause or not accepting referrals. Participants had to navigate inconsistent information, sudden changes to service delivery, new layers of triage and increased gatekeeping. Gatekeepers (i.e., receptionists, nurses, doctors and automated online/telephone systems) denied access or referred onward, depending on availability and the service sought. Typically, walk-in or same-day services were replaced with triage and appointment systems. The increased need for forward planning was a barrier to several participants, particularly those with children or other caring responsibilities for whom service flexibility facilitated access. Those requiring multiple healthcare interactions (e.g., STI treatment or antenatal services) experienced less difficulty once they were in the system. Participants with more healthcare knowledge and financial resources discussed how these minimised barriers to access. For example, one participant booked ‘a private scan that allowed partners to come in’ (M, 30–39).

In-person care was notably difficult to access, with one participant describing it as ‘impossible’. Some narrated ‘hitting a brick wall’ that stopped them from accessing services. Others described getting stuck in bureaucratic circles that delayed or prevented their healthcare access. One participant described being shunted like a ‘tennis ball between these two departments (the GP and midwifery)’ (M, 30–39). These barriers to service access were perceived by many as insurmountable, particularly for those seeking face-to-face appointments, LARC, asymptomatic STI testing or fertility treatments. Even with persistence and tenacity, not all were able to overcome barriers:

I couldn’t get in touch with the hospitals because they weren’t accepting referrals no more because of the coronavirus. With all the pains I was going through I couldn’t get any help whatsoever … you’re kind of just left to suffer. (F, 30–39)

Changes to services reduced participants’ sense of choice and autonomy, resulting in some accepting less preferred alternatives. Those seeking contraception for reasons other than preventing pregnancy (e.g., managing menopausal symptoms) felt their needs were deprioritised. Additionally, participants described how they were unable to continue SRH practices they saw as routine or positive, such as asymptomatic STI screening or cervical smear testing.

I'm totally frustrated because everybody should have the same right to be checked … they should have some period for people who just want to get [STI] tested. (F, 18–29)

### Experiencing telemedicine and ‘socially-distanced’ services

Twelve participants (or their partners) eventually attended the service they required. They described their experiences with services operating under COVID-19 protocols. Under such protocols, telemedicine and ‘socially-distanced’ healthcare had become a prominent part of service delivery, and services operated under reduced staffing.

These changes sometimes impacted on privacy. Some could not openly discuss their SRH needs during remote consultations within their home, while others discussed being expected to disclose sensitive information in settings that did not feel private (e.g., queuing outside the GP). Additionally, participants navigating telephone appointments discussed negative aspects, such as the difficulty of not knowing when a clinician would contact them and the challenge of having to restart the process if they missed their call-back. Some reported an unwelcome onus on the patient to follow up failed access attempts.

I was driving, I couldn't take the phone call and I think then I had to phone up the next day and I had to go through the same process. (F, 40–49)

Several participants reported that changes to the nature, pace and tone of clinical interactions detracted from their ability to address problems, ask questions or have spontaneous discussions. Participants felt rushed and that their healthcare needs were not well explained. A few felt that staff had less welcoming attitudes, aligning with formal requirements to minimise the risk of SARS-CoV-2 transmission. Many missed the reassurance of in-person appointments, in which they could communicate more easily:

I find them [telemedicine appointments] less personal … when you're on the phone it’s sort of not as easy to remember what you wanted to say, or what you need to ask. (F, 30–39)

The substitution of in-person appointments with remote consultations was experienced as less supportive for those more concerned about their health. Remote consultations were also difficult for people with accessibility needs, such as those with hearing difficulties.

For some, challenges were compounded by having to attend appointments alone without anyone to support them, ask questions or help remember information on their behalf. This concern was most prominent for those navigating fertility or antenatal/maternity services, an SRH need participants perceived as shared. The distress of lone attendance greatly impacted the pregnant partner:

All the times she went into hospital after we knew that there was something wrong, she was there alone, and it took a massive toll on her basically, and she is traumatised basically by that. (M, 30–39)

Lone attendance rules additionally left non-pregnant partners feeling disengaged, uncertain and less equipped to emotionally support their pregnant partner.

Some changes were experienced positively. A few participants noticed staff making extra efforts to be friendly and reassuring, to *‘make up for some of the other things that people are having to deal with*’ (F, 30–39). Many participants discussed the benefits of experiencing a combination of telemedicine and in-person care, which included shorter appointments and waiting times, less crowded waiting areas, and the convenience of postal delivery of medication such as contraception or antibiotic treatment.

### Attitudes toward the continuation of telemedicine

Many viewed the ongoing implementation of telemedicine, if used to complement in-person services, as potentially adding quality. One participant commented that *‘a lot of things could be resolved without needing a face-to-face consultation*’ (F, 40–49). Participants felt telemedicine could bring convenience to triaging, referrals, information giving and postal medication delivery:

I'd be more comfortable with a telephone appointment, as long as it was on a Friday and the children weren’t around … And if somebody could just send a prescription to my doctor, or to the pharmacy, so that I could collect my tablets there, then it would be fine. I don't really need to see anybody. (F, 40–49)

Telemedicine could allow people to save time and money normally spent on childcare or travel. Many appreciated being able to discuss sensitive health needs at home, a familiar and non-clinical environment. Telephone (but not video) appointments lessened perceived concerns about the stigma of accessing SRH services. Those critical of telemedicine worried that it would duplicate consultations or result in burdensome arrangements where calls were not returned and phone line staffing was variable. Several participants worried that explaining symptoms, physical examination and testing may be impossible remotely. Additionally, some highlighted that telemedicine would not work for them given their accessibility needs and requirements for privacy.

I prefer the contact, personal contact with somebody … I used to have phone anxiety in the past, so that’s why I prefer in person contact. (F, 18–29)

## Discussion

These findings complement previous research on how the COVID-19 pandemic has disrupted and impacted SRH,^12 13 17^ highlighting how unmet or delayed need might have resulted in worsened SRH outcomes, undermined reproductive choice, weakened preventive practices and increased distress over SRH. Participants faced barriers due to changing regulations regarding healthcare access, inconsistencies between and within services, the pausing of various services and difficulties navigating remote healthcare. Consequently, participants required health literacy and tenacity to access SRH services, which raises concerns about the amplification of pre-existing health inequalities given the social patterning of health literacy^18 19^ and existing barriers to SRH access.^20–23^

Telemedicine represents perhaps the most significant adaptation rapidly deployed by providers to mitigate the pandemic’s effects on healthcare. Our findings highlight how its ongoing deployment will require careful implementation to minimise barriers to access and enhance patient convenience. This study provides detailed qualitative evidence of the mixed picture concerning its acceptability and suitability. Corroborating wider research on adults’ experiences of SRH telemedicine,^3 5 24 25^ some Natsal-COVID participants held positive attitudes about its continuation, especially when complementing in-person care. This differs from findings among young people who hesitate to access remote SRH care.^13 26 27^ However, our participants were less satisfied with telemedicine during more sensitive and emotional consultations, highlighting limits to remote provision.^28^ This differs from findings on tele-abortion (an especially sensitive SRH service), with which participants reported satisfaction.^4–6^ Considering privacy and anonymity of SRH patients is crucial.^20 26 29^ Some participants worried that telemedicine might alert others in their household or community to their SRH needs. However, research has also highlighted the potential for telemedicine to promote privacy and anonymity in certain contexts such as abortion care.^24^ Given their varying needs and preferences, our data provide evidence that SRH patients should have the option of in-person, over-the-phone or video appointments to meet varying needs and preferences. This will require investment in training and equipment to ensure high-quality remote services.^30^

### Strength and limitations

We purposively interviewed participants who had tried but failed to access services and varied by age, gender, ethnicity and region. We included participants across Britain. However, a majority of these lived in England. This limits our ability to comment on differences in experiences across England, Scotland and Wales as each country has its own National Health Service system. Our enquiry followed a holistic framework of SRH spanning a wide range of services. Our approach provides insight into diverse experiences but limits our ability to comment in depth about specific SRH services or about services our participants did not have direct experience of, such as abortion and sexual assault services. Our study was unable to explore in depth the impacts of delayed access to specific services. Given the time between reporting of help-seeking and interview, we could not exclude potential for recall bias. This study aimed to highlight challenges and participants were recruited accordingly. Thus, the results likely under-represent positive experiences and downplay provider staff’s enormous efforts during this challenging time. Given participants’ digital recruitment, we may not have captured experiences of those without access to remote services, for example, due to language barriers, learning difficulties or socioeconomic factors. Finally, as with all qualitative research, our study draws on a small sample to capture a range of experiences of SRH access; it is not intended to be generalised or quantified.

## Conclusion and recommendations

Demand for services may increase due to a backlog of delayed help-seeking and the possibility of increased compensatory risk behaviours post-pandemic. Our study provides a general population perspective to complement service-user studies and quality improvement studies, offering recommendations for future practice derived from actual and potential patients in the community. Based on our qualitative data and in discussion with the study team and clinical colleagues, we set out a draft set of recommendations for consideration by service providers and policymakers (table 3). These recommendations link directly to the data and represent experience-learning from this unprecedented period to support a strong recovery, innovative and streamlined services in future, and resilience during future pandemics. However, implementing these recommendations must centre on the well-being of NHS staff, on whom the pandemic has taken a significant toll. Long-term adequate investment is crucial to safeguard staff and patient well-being.

**Table 3:** Recommendations

Service delivery aspect	Preliminary recommendations suggested from the data	Link to theme
Recovery phase	In encouraging re-engagement with services, reassure patients of the legitimacy of their needs and staff’s non-judgemental attitude. This applies particularly to those reporting risk behaviour during the pandemic. In the longer term – and once initial backlogs are cleared – targeted campaigns may be required to encourage re-engagement with cervical screening or asymptomatic STI testing and training for providers.	1
	Review gatekeeping functions established during the pandemic to ensure remaining triage systems do not create additional barriers for patient access.	2
Innovations to ‘usual practice’	Cautiously adopt telemedicine where it can enhance convenience and enable prompt testing, diagnosis and treatment responsiveness. It should be considered in addition to face-to-face services. Effort will be required to avoid telemedicine’s unnecessary bureaucratisation, duplication or the creation of added barriers to patient access. It will be crucial to avoid exacerbating inequalities in access and digital exclusion.	4
	Patient preferences for telephone, video or in-person consultation should be respected where possible, as needs, preferences and concerns vary.	3 and 4
	Ensure gatekeepers are aware of their role within a triage system so that they can supply patients with as much accurate, up-to-date and reassuring information as possible.	2
Service delivery during a pandemic	Prioritise cross-sector collaboration to avoid confusion over triage, particularly between pharmacies, GPs and specialist SRH providers.	2
	Set up agile and accessible mechanisms for sharing learning and good practice. The COVID-19 resources and regular meetings established by the British Association of Sexual Health and HIV (BASHH) are a good example of this.	ALL
	Ensure that information (eg, booking systems, opening hours) is continuously updated to avoid confusion for patients.	2
	Allow patients to be accompanied during pregnancy/antenatal services, during other emotionally demanding appointments, or to help with access needs (eg, language, disability, vulnerability) wherever possible.	3
	Establish safe ways to help patients feel comfortable in clinic to compensate for measures such as masks or socially distanced consultations. Small gestures (such as a warm greeting) may be ‘quick wins’ reducing stress both for the patient and professional and ensuring a more personable service.	3
	Continue with remote provision where possible, practicable and acceptable, ensuring staff are appropriately trained and supported to provide it.	4

## Data Availability

Data are available in a public, open access repository. Data are available upon reasonable request. Natsal-COVID qualitative interview data are available upon request from researchers. If interested, please contact kirstin.mitchell@glasgow.ac.uk. Quantitative data from this study are available from the UK Data Archive under safeguarded access, DOI: 10.5255/UKDA-SN-8865-1.
